# Outcomes and occurrence of post-operative pulmonary hypertension crisis after late referral truncus arteriosus repair

**DOI:** 10.3389/fcvm.2022.999032

**Published:** 2022-09-27

**Authors:** Yifan Zhu, Qi Jiang, Wen Zhang, Renjie Hu, Wei Dong, Hao Zhang, Haibo Zhang

**Affiliations:** ^1^Department of Cardiothoracic Surgery, Shanghai Children’s Medical Center, Shanghai, China; ^2^Shanghai Jiao Tong University School of Medicine, Shanghai, China

**Keywords:** truncus arteriosus, pulmonary hypertension crisis, late referral, congenital heart surgery, pediatrics

## Abstract

**Background:**

Truncus arteriosus (TA) is a rare congenital heart disease with a high rate of early mortality. The occurrence of post-operative pulmonary hypertension crisis (PHC), known to be a common and life-threatening complication, increases due to the irreversible development of pulmonary vascular resistance with age. We sought to figure out the risk factors for PHC and describe the surgical outcomes of TA patients with late referral (repair <1 month excluded).

**Materials and methods:**

We retrospectively reviewed patients after TA repair between 2009 and 2021 at Shanghai Children’s Medical Center. The occurrence of PHC was defined according to post-operative Pp/Ps ≥ 1 and clinical manifestations. Risk factors for PHC and mortality were conducted by multivariable analysis.

**Results:**

A total of 98 patients were treated, including 55 males and 43 females. The median age at repair was 121 (69, 245) days. Post-operative PHC occurred in 22 (22.4%) patients with a median age of 186 (122, 293) days. By multivariable analysis, patients with the sum of Z-score of pre-operative bilateral pulmonary artery (PA) diameters (OR: 1.6, 95% CI: 1.2–2.3, *P* = 0.01) was more likely to experience PHC. Longer CPB duration contributed to early death (OR: 1.0, 95% CI: 1.0–1.0, *P* = 0.01). Total survival at 10 years was 81.4%. In 4.5 (2.9, 7.5) years of follow-up, twenty-six patients received 30 reinterventions. Valved reconstruction of RVOT most predicted reinterventions (OR: 4.2, 95% CI: 1.4–13.0, *P* = 0.01).

**Conclusion:**

Surgical repair of TA patients with late referral has resulted in comparatively favorable early and mid-term outcomes. PHC occurred more commonly in patients with overextended bilateral PA pre-operatively. Meanwhile, valved reconstruction of RVOT would more likely lead to early reintervention.

## Introduction

Persistent truncus arteriosus (TA), known as a common arterial trunk, is an uncommon and complicated congenital heart disease that was well-described by Collett and Edwards (1) and further classified by Van Praagh (2). The single arterial trunk produces a chronic left-to-right shunt, which exposes pulmonary circulation to systemic circulation pressure. This condition results in irreversible and progressive pulmonary vascular obstructive disease as the patient ages. Thus, neonatal surgical treatment is recommended.

Early surgical procedures including pulmonary banding showed suboptimal clinical outcomes. Radical surgical operation using extracardiac conduit or autologous tissue to connect the right ventricle to the pulmonary artery achieved great early and long-term outcomes and was gradually accepted as the first choice. In recent decades, repair of TA yielded better survival and hemodynamic results in the neonatal period with improved surgical techniques, myocardial protection strategy, and intensive care.

In developing countries, however, patients presenting to the hospital for TA repair were older due to the lag in prenatal and postnatal diagnosis caused by unbalanced economic development in various regions (3). Late referral increased the incidence of post-operative pulmonary hypertension crisis (PHC) because of higher hazard of pulmonary vascular resistance. And this condition, presumed to be a significant risk factor influencing perioperative mortality, remained to be accurately clarified especially in patients without cardiac catheterization. Can we find a way to estimate the risk of occurrence of PHC in TA through non-invasive imaging methods pre-operatively? Not to accurately assess, but to correlate. In this study, we reviewed 13 years of experience with 98 cases, seeking to find the risk factors for the incidence of post-operative PHC and to describe the outcomes of patients after TA repair at a large single institution.

## Patients and methods

### Ethics statement

This is a retrospective, single-center study. The study was approved by the Institutional Review Board of Shanghai Children’s Medical Center Affiliated with Shanghai Jiao Tong University School of Medicine (approval number SCMCIRB-K2022070), which waived the need for consent from individual patients.

### Patients

From January 2009 to December 2021, all patients who received primary repair of TA at Shanghai Children’s Medical Center were included. The patients were excluded if they were (1) operated at the neonatal period; (2) performed with palliative procedures; (3) received the initial operation at other institutions; (4) diagnosed with hemi-truncus (anomalous origin of one pulmonary artery from the aorta).

### Pre-operative characteristics

All patients underwent transthoracic echocardiography. At our center, 80.1% of patients underwent primary cardiac computed tomography (CT) or cardiac magnetic resonance imaging (MRI), and patients older than 6 months underwent pre-operative cardiac catheterization. Pre-operative diameter of bilateral pulmonary artery (PA), both at the middle and ostia, was mostly gained from CT. Data obtained from pre-operative echocardiography included the pathologic anatomy and function of the truncal valve, the origin and anatomic features of aberrant bilateral PAs, aortic arch, valvular function, and concomitant cardiac anomalies. The pulmonary artery systolic pressure (PASP) was estimated by the peak flow velocity of tricuspid regurgitation (TR) and the mean pressure of the right atria (4). Calculated Z-scores of the diameter of the truncal valve and bilateral PAs were used to normalize the transthoracic echocardiography measurements to the patients’ body size (5).

### Surgical techniques

All patients underwent median sternotomy and establishment of cardiopulmonary bypass (CPB). After fully dissecting and exposure of the aorta and bilateral PAs, right ventricular outflow tract (RVOT) reconstruction was performed using autologous tissue and extracardiac conduit. Patients with enough length of the main pulmonary artery (MPA) often received a pull-down approach and were directly anastomosed to right ventricular incision. For patients with type A2, a cylindrical segment incorporating the bilateral PAs was sleeve-resected from the truncal artery. Then, the frontier wall of the cylinder was cut off and a side-to-side anastomosis was performed to form the posterior wall of RVOT. If necessary, the left auricle and a portion of the aorta transected together could be used as the posterior wall of the RVOT. A hood patch was anastomosed to the anterior wall of RVOT to avoid distortion. Or, a Gore-tex conduit with or without handmade valve and Bovine Jugular Vein (BJV) conduit were chosen. Truncal valve repair was needed if patients suffered from significant regurgitation. It included resuspension, commissure suturing, and removing the prolapsed valve. Full-thickness plication or excision of part of the aortic wall was needed in patients with dilated aortic roots. Early replacement of the primary aortic valve was not recommended. Lecompte maneuver was performed if the right PA was compressed or distorted during the operation.

### Definitions

The type of TA was classified according to Van Praagh (A1–A4) (2). The late referral was defined as not receiving initial surgery during the neonatal period. Truncal valve insufficiency (TVI) was graded qualitatively as none/trivial, mild, mild-moderate/moderate, and moderate-severe/severe. Significant valvular regurgitation was defined as moderate or greater. The diameter of bilateral PAs size was measured at the middle point. Early mortality was defined as those who died before hospital discharge or within 30 days after the operation. Post-operative PHC was assessed by Pp/Ps > 1 or severe clinical manifestation in the operation room or intensive care unit (ICU): sudden hypoxia, decreased cardiac output, hypotensive shock, and history of cardiopulmonary resuscitation. A combination of bosentan and iloprost solution for inhalation was the most commonly used for prophylactic treatment for post-operative PA hypertension. RVOT-related reinterventions included surgical reconstruction and percutaneous stent implantation or balloon dilation. Residual peak flow velocity in PA > 3 m/s or with obvious clinical symptoms necessitated reintervention during follow-ups at our institution.

### Statistical analysis

Data were analyzed using SPSS software, version 26.0 (IBM Corp., Armonk, NY, USA). Continuous variables were presented as median (25th and 75th percentiles). Categorical variables were expressed as frequency and percentages. Comparison between groups was performed using the Mann–Whitney *U* test or Fisher’s exact test or chi-square test for categorical variables. A receiver operating characteristics (ROC) curve was used to identify the optimal cutoff point. Time-to-event distribution was determined using the Kaplan–Meier method. The difference in time-to-event distribution was determined using the log-rank test. Risk factors for PHC and early mortality were analyzed using logistic regression. Risk factors for first reintervention were compared using COX proportional hazard modeling. Variables with a *P*-value < 0.10 calculated by univariate analysis and variables with clinical significance were subordinated to the multivariable analysis. *P*-value < 0.05 were considered statistically significant.

## Results

A total of 98 patients were enrolled in this study from 2009 to 2021. Baseline characteristics are summarized in Table 1. Of note, twenty-two patients suffered post-operative PHC and 76 patients did not. The median age at surgery in the PHC group was 186 (122, 293) days, and the age in the group without PHC was much smaller (*P* = 0.013). Neonatal repair was excluded from this study. The median weight at surgery was 5.0 (4.3, 6.6) kg. Neither weight, sex, body surface area (BSA), truncal type, truncal valve anatomy, size, TVI, left ventricular ejection fraction (LVEF) nor pre-operative significant TR reached a significant difference between groups with or without PHC (*P* > 0.05). The systemic systolic pressure was higher in the group with PHC (*P* = 0.027). Interestingly, the size of bilateral PA and the sum of the Z-score of PA diameter were all larger in patients with PHC (*P* < 0.05). Cardiac catheterization was performed on twenty-eight patients, of whom mean pulmonary artery pressure (mPAP) was 63.0 (58.0, 66.0) mmHg in the group with PHC and 39.0 (29.5, 52.0) mmHg in the group without PHC, respectively (*P* < 0.05). Pre-operatively, mechanical ventilation was required in 13 patients.

**TABLE 1:** Patients’ characteristics before surgical repair of truncus arteriosus.

	Overall	With PHC	Without PHC	*P*-value
Number	98	22	76	
Age (d)	121 (69, 245)	186 (122, 293)	105 (60, 205)	0.013*
Weight (kg)	5.0 (4.3, 6.6)	6.3 (4.7, 7.7)	5.0 (4.3, 6.4)	0.106
Male	55 (56.1%)	10 (45.4%)	45 (59.2%)	0.331
BSA (m^2^)	0.28 (0.24, 0.34)	0.31 (0.26, 0.37)	0.27 (0.23, 0.32)	0.068
Truncal type				0.682
A1	46	11	35	
A2	41	10	31	
A3	6	0	6	
A4	5	1	4	
Truncal valve anatomy				0.211
Bicuspid	18	4	14	
Tricuspid	68	14	54	
Quadricuspid	12	4	8	
Truncal valve diameter (cm)	1.5 (1.3, 1.9)	1.8 (1.5, 2.2)	1.5 (1.3, 1.9)	0.092
Truncal valve Z-score	6.8 (5.7, 7.6)	7.4 (5.6, 8.0)	6.8 (5.7, 7.5)	0.297
Truncal valve insufficiency				0.785
None/trivial	32	7	25	
Mild	52	11	41	
Mild-mod/mod	11	4	7	
Mod-severe/Severe	3		3	
Pre-operative LVEF (%)	68.5 (64.0, 72.8)	67.0 (61.5, 72.0)	68.7 (64.7, 73.2)	0.357
Pre-operative systemic systolic pressure (mmHg)	85.0 (78.0, 92.0)	87.0 (83.7, 96.3)	84.0 (77.0, 89.0)	0.027*
Pre-operative catheterization	34 (34.7%)	10 (45.5%)	24 (31.6%)	0.520
Pre-operative RPA diameter	0.7 (0.6, 1.0)	0.9 (0.8, 1.1)	0.7 (0.6, 0.9)	0.002*
Pre-operative LPA diameter	0.6 (0.5, 0.8)	0.8 (0.6, 0.9)	0.6 (0.5, 0.7)	0.019*
Calculated Z-scores				
RPA diameter	1.7 (0.6, 2.9)	2.8 (1.6, 3.5)	1.4 (0.4, 2.5)	0.004*
LPA diameter	1.3 (−0.2, 2.5)	2.0 (1.2 2.9)	1.2 (−0.3, 1.9)	0.023*
Z-RPA + Z-LPA	3.0 (0.5, 4.7)	4.7 (3.7, 5.9)	2.4 (0.3, 4.2)	0.002*
Pre-operative sig-TR	18 (18.4%)	5 (22.7%)	13 (17.1%)	0.550
Pre-operative history of pneumonia or ventilation	13 (12.8%)	2 (9.1%)	11 (14.5%)	0.321

PHC, pulmonary artery crisis; BSA, body surface area; LVEF, left ventricular ejection fraction; RPA, right pulmonary artery; LPA, left pulmonary artery; TR, tricuspid regurgitation.

*P < 0.05.

Operation data are listed in Table 2. The median CPB time was 165 (124, 195) min in the group with PHC and 134 (114, 165) min in the group without PHC (*P* = 0.027). The median aortic cross-clamp time was longer in the group with PHC while did not reach statistical significance (*P* = 0.088). There were 38 patients (38.8%) receiving MPA pull-down and anastomosed to right ventricle directly. Of note, seven patients used left auricle as posterior wall of RVOT. In the remaining 60 patients (61.2%), 35 used BJV conduits and 25 used Gore-tex conduits of which nearly half were non-valved. Truncal valvuloplasty was performed in 14 patients (14.3%). Coronary artery anomaly (CAA) was present in 26 patients (26.5%). None of above characteristics was statistically different between two groups (*P* > 0.05). Median MPA diameter, referred to as extracardiac conduit diameter and size of pulmonary artery probe, was 12.0 (10.3, 15.0) mm and 41.2 (35.2, 47.6) mm/m^2^ indexed to BSA. A total of thirty-two patients suffered from delayed sternal closure. Other surgical approaches are also shown in Table 2.

**TABLE 2:** Operation data.

	Overall	With PHC	Without PHC	*P*-value
CPB time (min)	143 (117, 168)	165 (124, 195)	134 (114, 165)	0.027*
ACCT time (min)	88 (72, 110)	98 (85, 118)	88 (69, 109)	0.088
CAA	26 (26.5%)	8 (36.4%)	18 (23.7%)	0.196
IAA repair	7 (7.1%)	1 (4.5%)	6 (7.9%)	0.655
Truncal valve repair	14 (14.3%)	4 (18.2%)	10 (13.2%)	0.582
RVOT reconstruction				0.546
Extracardiac conduit	60	15	45	
Autologous tissue	38	7	31	
Neo Pulmonary valve				0.116
With	62	18	44	
Without	36	4	32	
MPA diameter (mm)	12.0 (10.3, 15.0)	13.0 (11.4, 14.5)	12.5 (10.0, 15.0)	0.438
MPA/BSA (mm/m^2^)	41.2 (35.2, 47.6)	42.1 (37.1, 45.1)	40.4 (34.4, 48.3)	0.926
Delayed sternal closure	32 (32.7%)	11 (50%)	31 (40.8%)	0.310
Diaphragm paralysis	7 (7.1%)	2 (9.1%)	5 (6.6%)	0.446
Fenestrated VSD or PFO	14 (14.3%)	4 (18.2%)	10 (13.2%)	0.263
Lecompte maneuver	7 (7.1%)	1 (4.5%)	6 (7.9%)	0.791
ECMO	1 (1.0%)	1	0	

PHC, pulmonary artery crisis; CPB, cardiopulmonary bypass; ACCT, aortic cross-clamp time; CAA, coronary artery anomaly; IAA, interrupted aortic arch; RVOT, right ventricular outflow tract; MPA, main pulmonary artery; BSA, body surface area; VSD, ventricular septal defect; PFO, patent foramen ovale; ECMO, extracorporeal membrane oxygenation. *P < 0.05.

### Post-operative outcomes and risk factor analysis

**FIGURE 1:**
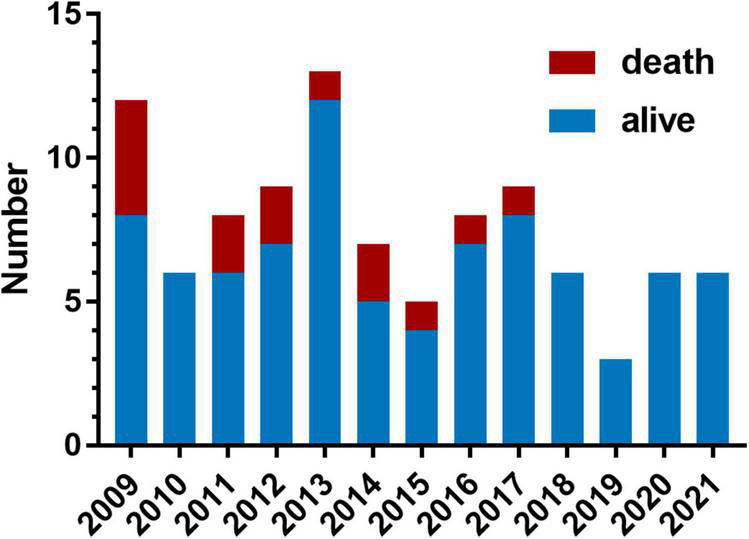
Variation across years in the number of patients who underwent truncus arteriosus (TA) repair and the early mortality. Each bar contains the number of patients who suffered operative mortality (red portion) and the number of patients who survived (blue portion).

**FIGURE 2:**
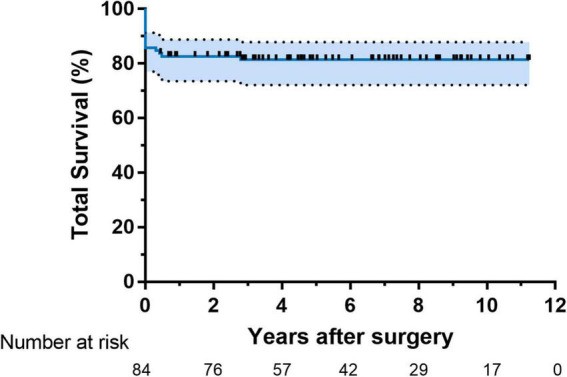
Kaplan–Meier analysis of time-related total survival.

The distribution of surgery numbers by era depicted in Figure 1 shows that surgical repairs before 2015 were associated with relatively increased early mortality compared with operations performed in later eras (20% vs. 7.0%, *P* < 0.05). Median ICU stay was 9 (7.3, 13.8) days in the group with PHC and 7 (6, 7) days in the group without PHC, respectively. There was no significant difference between patients with or without PHC in early outcomes except for numbers of patients subjected to significant TR post-operatively (*P* = 0.009) (Table 3). Both of the bilateral PA sizes decreased apparently compared with the pre-operative diameter of branch PAs (*P* < 0.05). Early mortality was 14.2% (*n* = 14). Late death occurred in four patients. Total survival at 1 and 10 years was 82.6% and 81.4% in patients within follow-ups (Figure 2). Low cardiac output, multiple organ failure, and malignant arrhythmia were responsible for the early death of the remaining cases of death. Multivariable analysis revealed the relationship between early mortality and longer CPB time (OR 1.0, 95% CI: 1.0, 1.0, *P* = 0.01) (Table 4). Notably, many risk factors, previously deemed to be related to mortality such as low body weight, CAA, interrupted aortic arch (IAA), pre-operative LVEF, significant TVI, and PHC did not reach statistical significance in this study.

**TABLE 3:** Early outcomes.

	Overall	With PHC	Without PHC	*P*-value
ICU stay (d)	7 (6, 12)	9 (7.3, 13.8)	7 (6, 12)	0.173
LVEF (%)	67.9 (62.4, 73.4)	69 (63, 76)	67 (62, 73)	0.150
RPA diameter (cm)	0.6 (0.5, 0.7)	0.7 (0.6, 0.8)	0.6 (0.5, 0.7)	0.071
LPA diameter (cm)	0.6 (0.5, 0.7)	0.7 (0.6, 0.8)	0.6 (0.5, 0.7)	0.065
MPA diameter (cm)	1.1 (0.9, 1.3)	1.2 (1.0, 1.4)	1.1 (0.8, 1.3)	0.121
Median PASP (mmHg)	56.7 (41.6, 69.1)	61.2 (51.2, 69.7)	52.5 (40.5, 69.0)	0.368
Significant TR	11 (11.2%)	5 (22.7%)	6 (7.9%)	0.009*
Significant PI	34 (34.7%)	8 (36.4%)	26 (34.2%)	0.516
Significant AI	7 (7.1%)	2 (9.1%)	5 (6.6%)	0.384
Number of drugs used				0.120
Bosentan	34	12	22	
Treprostinil	10	3	7	
Iloprost	32	13	19	
Vardenafil	3	2	1	
Discharge medication	28 (28.6%)	11 (50%)	17 (22.4%)	0.875

PHC, pulmonary artery crisis; ICU, intensive care unit; LVEF, left ventricular ejection fraction; RPA, right pulmonary artery; LPA, left pulmonary artery; PASP, pulmonary artery systolic pressure; MPA, main pulmonary artery; PI, pulmonary insufficiency; AI, aortic insufficiency; TR, tricuspid regurgitation. *P < 0.05.

**TABLE 4:** Univariable and multivariable analysis for risk factors of early mortality by logistic regression.

	Univariable		Multivariable	
	OR (95% CI)	*P*-value	OR (95% CI)	*P*-value
IAA	4.6 (0.9, 23.6)	0.06	0.8 (0.1, 9.3)	0.26
Weight at surgery	0.7 (0.5, 1.0)	0.08	0.7 (0.4, 1.1)	0.06
CPB time	1.0 (1.0, 1.0)	0.03*	1.0 (1.0, 1.0)	0.01*
Occurrence of PHC	12.7 (3.0, 55)	0.14	1.2 (0.2, 6.6)	0.88
Delayed sternal closure	3.5 (1.1, 11.3)	0.04*	1.2 (0.3, 5.8)	0.35
Pre-operative LVEF	0.9 (0.8, 1.0)	0.04*	0.9 (0.8, 1.0)	0.14

IAA, interrupted aortic arch; CPB, cardiopulmonary bypass; PHC, pulmonary artery crisis; LVEF, left ventricular ejection fraction; OR, odds ratio; CI, confidence interval. *P < 0.05.

**FIGURE 3:**
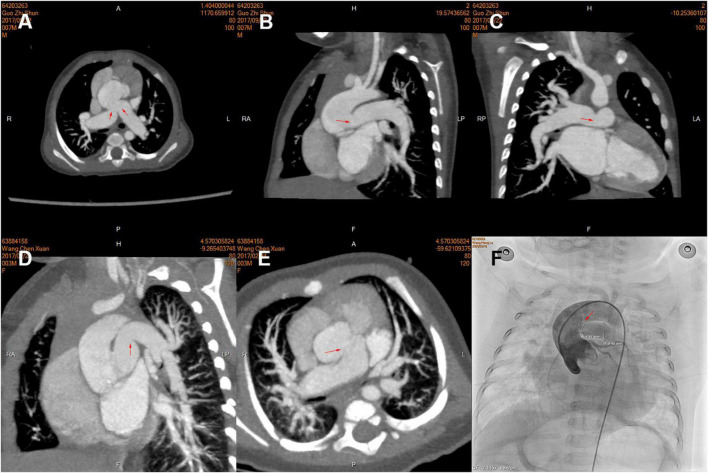
Pre-operative computed tomography (CT) angiography and cardiac catheterization showing branch pulmonary artery (PA) morphology of the different types of truncus arteriosus (TA), including the proximal and distal in three patients. **(A–C)** Type A2; **(D,E)** Type A1; **(F)** Type A3. The opening of branch PAs is labeled by red arrows.

Pulmonary hypertension crisis affected twenty-two (22.4%) patients, all of which occurred in hospital; six patients died of post-operative PHC in the hospital, and one of them received ECMO after operation. We found patients with PHC often had larger bilateral PA size (indexed to Z-score) using logistic regression (Table 5) or both by imaging results in different truncal types (Figure 3). Thus, we identified the sum of Z-score of bilateral PAs > 4 (area under curve: 0.75, 95% CI: 0.61–0.91) as the optimal cutoff point for prediction of PHC by ROC. As expected, PHC occurred in 16 (51.6%) of the 31 children with a sum of Z scores of PAs greater than 4, while PHC occurred in only 6 (8.9%) of the remaining 67 children with Z scores less than 4. In addition, in patients with a sum of Z scores > 4, the mPAP was larger (54.9 ± 10.1 mmHg vs. 41.9 ± 15.6 mmHg, *P* = 0.04).

**TABLE 5:** Univariable and multivariable analysis for risk factors of post-operative PHC occurrence by logistic regression.

	Univariable		Multivariable	
	OR (95% CI)	*P*-value	OR (95% CI)	*P*-value
Age	1.0 (0.9, 1.0)	0.54		
Weight at surgery	1.1 (0.9, 1.3)	0.27		
IAA	0.6 (0.1, 4.9)	0.59		
Significant TVI	1.6 (0.4, 7.2)	0.51		
Significant MR	2.8 (0.6, 13.8)	0.10	2.4 (0.3, 16.5)	0.39
Truncal valve diameter	2.5 (0.6, 10.6)	0.18	2.3 (0.2, 34.9)	0.53
The sum of Z-score of RPA and LPA diameter	1.5 (1.2, 2.0)	0.001*	1.6 (1.2, 2.3)	0.01*
LVEF	0.9 (0.9, 1.0)	0.32		
Systemic systolic pressure	1.1 (1.0, 1.1)	0.02*	1.1 (0.9, 1.2)	0.18
CAA	2.4 (0.9, 6.6)	0.09	5.1 (0.9, 27.6)	0.06
Conduit diameter	1.1 (0.9, 1.3)	0.56		
CPB time	1.0 (1.0, 1.0)	0.01*	1.0 (0.9, 1.0)	0.24
ACCT time	1.0 (1.0, 1.0)	0.03*	1.0 (0.9, 1.0)	0.78
Valved RVOT reconstruction	3.7 (1.1, 12.1)	0.03*	1.1 (0.2, 7.2)	0.94
Fenestrated VSD or PFO	1.6 (0.5, 6.0)	0.44		
Lecompte maneuver	0.6 (0.1, 4.9)	0.59		
Truncal valve repair	1.2 (0.3, 4.8)	0.81		
Delayed sternal closure	1.6 (0.6, 4.2)	0.32		

PHC, pulmonary artery crisis; IAA, interrupted aortic arch; TVI, truncal valve insufficiency; MR, mitral regurgitation; RPA, right pulmonary artery; LPA, left pulmonary artery; LVEF, left ventricular ejection fraction; CPB, cardiopulmonary bypass; ACCT, aortic cross-clamp time; CAA, coronary artery anomaly; RVOT, right ventricular outflow tract; VSD, ventricular septal defect; PFO, patent foramen ovale; OR, odds ratio; CI, confidence interval. *P < 0.05.

### Follow-ups and reinterventions

The median follow-up time was 4.5 (2.9, 7.5) years. A total of eighty-four patients were discharged from the hospital. At a median age of 1.0 (0.6, 4.8) years with a median follow-up period of 0.5 (0.3, 2.2) years after the initial operation, four patients (4.7%) died; 3 of whom died of low cardiac output secondary to RVOT reintervention and 1 died after mechanical aortic valve replacement. At a median time of 3.4 (1.5, 5.3) years, twenty-six patients received 30 RV-PA related reinterventions, including 4 balloon dilations and 26 RVOT reconstructions.

**FIGURE 4:**
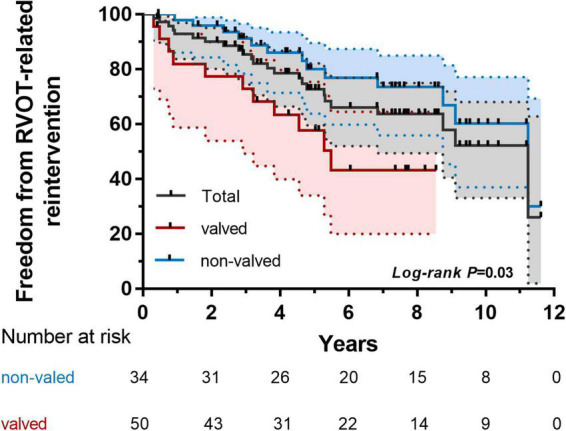
Kaplan–Meier analysis of freedom from right ventricular outflow tract (RVOT)-related reintervention of subgroups (valved and non-valved).

We found that MPA size indexed to BSA, autologous patch or extracardiac conduit, and significant pulmonary insufficiency (PI) were all independent of further reintervention (*P* > 0.05). Valved reconstruction of RVOT was independently associated with RVOT-related reintervention by COX proportional hazard modeling (Table 6). Patients with a neo-pulmonary valve achieved suboptimal freedom from RVOT-related reintervention (Figure 4).

**TABLE 6:** Multivariable Cox proportional hazard modeling for risk factors of RVOT-related reinterventions.

	HR (95% CI)	*P*-value
Valved MPA	4.2 (1.4, 13.0)	0.01*

MPA, main pulmonary artery; RVOT, right ventricular outflow tract; HR, hazard ratio; CI, confidence interval. *P < 0.05.

## Discussion

Since the first repair of TA with a valved conduit was successfully performed by McGoon and Rastelli (8), the single-staged surgery tended to be done in the early period of life. However, hospital mortality remained high only next to the Norwood procedure (6). Thus, neonatal repair of TA was recommended due to the risk of aggravation of pulmonary vascular obstruction with age. Patients who received TA repair achieved gradually improved early outcomes and excellent long-term survival if getting through the first year after surgery in recent decades (7, 9, 10). Unfortunately, due to the unbalanced medical development and distribution in developing countries, patients with TA referred to our hospital tend to be older than 1 month. Missing the optimal treatment window makes initial surgical treatment more challenging, but this study still achieved satisfactory early results and similar long-term survival benefits.

Although the early mortality at our center was not satisfactory, it has fallen to 7% in the past 5 years because of our emphasis on the prediction and management of PHC. The median age for surgical repair of TA was relatively high due to late referral to the hospital, which was also suggestive of existence of pulmonary vascular obstruction induced by prolonged exposure of pulmonary circulation to systemic circulation in older children. Ebert and colleagues (11) also pioneered TA repair beyond the first 6 months and achieved better survival. However, we did not find that elder age at repair increased the hazard of early death in this study. Elective repair at several months of age can also be advocated for some reasons: (1) clinical conditions without IAA, severe TVI, excessive pulmonary flow, severe pneumonia, or heart dysfunction can better withstand the operation; (2) physiological PA hypertension in the neonatal period might interfere with a surgeon’s choice of approach. Early mortality was further improved by the introduction of heart protection protocols, perfusion method, better intensive care, and use of drugs to reduce the pulmonary vascular resistance. Predictors, reported previously (12–15), such as low body weight, IAA, and repair of TVI, were no longer difficult to deal with. Instead, a longer duration of CPB was proven to be associated with early death. Similar results were demonstrated by Mastropietro and colleagues (16). The extension of parallel circulation time often accounted for longer CPB time, which resulted from swollen heart, arrhythmia, pulmonary atelectasis, effusion of blood at the anastomotic site, and low cardiac output. Of course, complicated malformations or the surgeon’s proficiency may also be involved.

We are more concerned about how to predict the incidence of PHC in patients who have reached a certain age in a convenient and non-invasive way. The occurrence of PHC was generally within 1–2 days after surgery, some even in the operating room. Few studies focused on the factors of life-threatening PHC during the post-operative period. We found that 22.4% of patients were subjected to PHC after the initial operation with large size of bilateral PAs. It was reported that the size of MPA was significantly correlated with PA pressure in common children (17, 18). MPA expands with PA pressure, just as inferior vena cava changes with TR. Because of the absence or hypoplasia of MPA in children with TA before surgery, we used the size of bilateral PAs instead. Besides, the branch PAs were much closer to the lungs, which were more sensitive to PA pressure. We used Z-scores instead of the diameter of branch PAs because of the inherently different development of right and left PAs. This hypothesis was first proposed and described in our previous study of patients with hemi-truncus (19).

First, dilated PAs were often an indicator of pre-operatively high PA pressure, which resulted from a combination of genetic predisposition, intrinsic characteristics of the vessel, and an adaptive response to elevated PA pressure. In patients with TA, bilateral pulmonary high-flow perfusion, induced by large PA size, was mainly due to the connection between PA and systemic circulation. The pre-operative mPAP in patients with larger PA size (sum of Z-score of PAs > 4) was also much higher. Prifti and colleagues also attributed it to increased blood flow into the lungs (20). Moreover, early PA hypertension is often reversible after surgery in patients younger than 6 months, which would become stubborn over time and lead to more exaggerated PA expansion. In patients older than 6 months, pre-operative median mPAP was higher (50 mmHg) and sum of Z-scores was larger (*Z* = 3.73). Of note, we found there was often a reactive constriction of the PA at the ostia of ascending aorta by CT. In addition, it was more common in dilated PAs, as shown in Figures 3A–C. We used the ostia/middle ratio of branch PA to describe the degree of constriction. The ratios of RPA and LPA were 0.95 and 0.93 in Group (sum of Z-score > 4) and 1.2 and 1.2 in the group (sum of Z-score < 4). In addition, median age of the former group was also older. In pathophysiology, pulmonary arteriole intimal fibrosis hyperplasia and PA dilatation often indicate the presence of late irreversible pulmonary hypertension. All of these could explain why these patients were at higher risk hazard of occurrence of post-operative PHC. More specific characteristics in morphology and hemodynamics should be of concern and investigated stepwise. We do not advocate the use of sum of Z-scores of PAs to screen for PA hypertension in children with TA. We recommend that it can be used to alert surgeons to this possibility so that further examinations and approaches may be performed if necessary.

Meanwhile, there was a high reintervention rate of RVOT or branch PA for stenosis and conduit failure. The largest multicenter comparative study reported a 10-year freedom from reintervention rate of 25% and a median time to reintervention of 4.3 years (13), which matched our experience. Logically, smaller conduits tended to lead to early reintervention due to growth potential in young infants, which was already recognized by Sinzobahamvya and colleagues (21). It did not reach statistical significance if indexed to BSA on account of modified surgical techniques and choosing MPA size carefully in this study. In other institutions, MPA/BSA = 50 mm/m^2^ was recommended in case of major adverse cardiovascular events that were revealed by Mastropietro and colleagues (16). We also did not find a significant disparity between autologous tissue and extracardiac conduit in RVOT reconstruction. Hooding and wedge-shaped trimming of the proximal MPA enabled us to avoid early reintervention resulting from distortion or mismatching of the MPA to right ventricle.

The presence of a pulmonary valve at RVOT suggested the high risk of reintervention in this study. Calcification, crispatura, and stiffness of the pulmonary valve would eventually occur due to the nature of the valvular material. RVOT stenosis was responsible for 70.4% (19/27) of reinterventions. Even so, we would rather take the risk of a high reintervention rate to form a pulmonary valve in order to avoid the possibility of poor early outcomes as much as possible. Danton and colleagues (22) pointed out that a valved conduit was inevitable to protect the right ventricular function and prevent early PHC. For most patients, a valved conduit or a hand-made valve was necessary at the RVOT. However, for small infants, the absence of a pulmonary valve will not affect their survival benefit, which may also delay the time to reintervention (23). Because the severity of pulmonary vascular obstructions was not high in infants who received early surgical treatment, the right ventricular was able to tolerate mild pulmonary insufficiency. In addition, there was no significant difference in the degree of long-term pulmonary regurgitation with or without pulmonary valve. The non-valved treatment strategy was selected to reduce the incidence of reintervention caused by residual stenosis or valvular dysfunction in some infants. A pulmonary valve reconstruction or implantation could be performed when the infant grows up. There was always a pendulum between the choice of reducing the reintervention rate and preservation of right ventricular function.

## Limitations

There are several limitations in this study due to its retrospective nature. Results may be affected by selection bias. The choice of size and kind of MPA varied due to surgeons’ experience. The commercialized BJV conduit has only been widely popularized in recent years in our country, which may lead to bias in the selection of RVOT types in different eras. More detailed characteristics of the branch PAs need to be further analyzed. The degree of ultrasonic indicators may slightly vary among several cardiac sonographers. The timing of referral for reintervention for those asymptomatic patients and treatment protocols may vary among different surgeons for personal preference and the acceptance of parents. In addition, further follow up is needed.

## Conclusion

Hospital mortality and occurrence of post-operative PHC were relatively not rare in the TA entity. At our institution, we achieved decreased early mortality by era and excellent late survival in patients with a late referral. A pre-operative larger sum of the Z-score of PAs was an independent predictor for the post-operative occurrence of PHC. We are not trying to screen PHC by the indicator but to provide a reference for subsequent treatment. Valved reconstruction of MPA contributed to reintervention, which can be well accepted.

## Data availability statement

The original contributions presented in this study are included in the article/supplementary material, further inquiries can be directed to the corresponding authors.

## Ethics statement

Written informed consent was obtained from the minor(s)’ legal guardian/next of kin for the publication of any potentially identifiable images or data included in this article.

## Author contributions

YZ and QJ: conception, collection, analysis, and drafting. WZ and RH: revision and visualization. WD: revision and supervision. HZ and HBZ: funding, revision, supervision, and final approval. All authors have approved the final version.
